# On *p*-Values and Statistical Significance

**DOI:** 10.3390/jcm12030900

**Published:** 2023-01-23

**Authors:** Stefanos Bonovas, Daniele Piovani

**Affiliations:** 1 Department of Biomedical Sciences, Humanitas University, Pieve Emanuele, 20072 Milan, Italy; 2 IRCCS Humanitas Research Hospital, Rozzano, 20089 Milan, Italy

At the beginning of our research training, we learned about hypothesis testing, *p*-values, and statistical inference. In hypothesis testing, we first set up a null hypothesis and an alternative hypothesis. For example, if we are interested in comparing proportions between two groups, then the null hypothesis would be stated as “the proportions are the same” and the alternative hypothesis as “the proportions differ” between the two groups. We then gather data to test the veracity of the null hypothesis. In particular, the data allow us to calculate the *p*-value, which is defined as “*the probability under a specified statistical model that a statistical summary of the data would be equal to or more extreme than its observed value*” [1], and practically reflects the degree of data compatibility with the null hypothesis. Conventionally, if the *p*-value is lower than the 0.05 significance level, we reject the null hypothesis and accept the alternative hypothesis. 

This methodology, often referred to as “null hypothesis significance testing”, originated in the early 20th century with the ideas of Fisher [2], Neyman, and Pearson [3]. Since then, the *p*-value has become the most frequently used statistic in the biomedical research field—a sine qua non for determining if a study finding is real or due to chance, if a new drug is effective or not, or if a health technology should be approved or denied by regulatory authorities. However, in recent years, there is a growing discussion that *p*-values and “statistical significance” (i.e., the classification of results as “significant” or “not significant” based on whether the *p*-value is lower than a certain cut-off threshold), albeit useful, have often been misused and misinterpreted in the biomedical field [4,5,6,7,8,9,10]. Moreover, the advent of Big Bata [11] will further exacerbate this problem. This article aims to draw readers’ attention toward the appropriate use and interpretation of *p*-values in clinical research. 

To begin with, let us assume that a single-center, randomized, double-blind, placebo-controlled trial is performed to determine if a new drug reduces mortality in 400 patients hospitalized with severe COVID-19 pneumonia, who are otherwise receiving standard-of-care. The occurrence of death at 28 days following randomization is the trial’s primary endpoint. The numbers of patients who died or survived at 28 days, in each of the two study groups, are given in Table 1 (hypothetical data for illustrative purposes).

Assuming that the particular study suffers no systematic error (bias) or confounding, we can employ significance testing or confidence interval (CI) methods to examine whether there is statistical evidence against the null hypothesis (i.e., that there is no difference between the active drug and placebo regarding mortality at 28 days). Using the data from Table 1, we can calculate a *p*-value that is higher than the cut-off threshold of 0.05 (i.e., *p*-value = 0.11). Equivalently, the Risk Ratio is 0.70 (i.e., showing a 30% lower risk of death in the active drug group compared to the placebo group), and the 95% CI includes the null value of one (i.e., Risk Ratio = 0.70, 95% CI: 0.45–1.09; Table 1). Can we conclude that there is “no difference” between the active drug and placebo regarding mortality at 28 days? The correct answer is “no”. We should not claim that there is “no difference”, or that the drug is “equally effective” to placebo just because a *p*-value is higher than an arbitrary threshold (e.g., 0.05 or 0.01), or because the null value falls within the confidence interval. A statistically non-significant result does not “prove” the null hypothesis [12]. This is the reason why the correct statement regarding hypothesis testing would be that “we failed to reject the null hypothesis” and not that “we accepted the null hypothesis”. In other words, if the current experiment did not provide enough evidence against the null hypothesis, it does not mean the null hypothesis is true! Failing to prove the contrary of a theory does not mean that the theory is correct (e.g., failing to prove the defendant is guilty does not necessarily mean that the defendant is truly innocent). Moreover, the confidence interval is wide (i.e., 95% CI: 0.45–1.09), indicating considerable uncertainty. While this may seem to be a semantic nuance, it is not, and it has very relevant consequences for decision making. Importantly, we should be very cautious when drawing conclusions, or making policy decisions because the “absence of evidence is not evidence of absence” regarding a difference or association [13]. 

Let us now assume that a subsequent multi-center, randomized, double-blind, placebo-controlled trial assessed the efficacy of the same experimental drug in 1700 patients hospitalized with severe COVID-19 pneumonia (Table 2; hypothetical data for illustrative purposes). 

Using the data from Table 2, we can calculate a *p*-value that is lower than the threshold of 0.05 (i.e., *p*-value = 0.001). The Risk Ratio is 0.70 (i.e., identical to that of the previous trial, and showing again a 30% lower risk of death in the active drug group), but the 95% CI now excludes the null value of one (i.e., Risk Ratio = 0.70, 95% CI: 0.57–0.87; Table 2). Can we claim that the two studies are in contrast because the latter had a statistically significant result (i.e., *p*-value < 0.05) while the first one did not? Again, the answer is “no”. We cannot make such a claim! This is another misunderstanding about *p*-values. Especially when the study is underpowered and/or of a small sample size (e.g., our first trial example), the *p*-value has a large variance across repeated samples (i.e., experiments conducted on different samples of the same population) [14]. This is why considering the *p*-value obtained in a study to be indicative of the likelihood of getting the same results in future studies, conducted on the same topic, is not correct.

It is wrong to claim that a statistically non-significant result shows “no association”, when the confidence interval includes clinically important decreases (or increases) in risk. Similarly, it is wrong to claim that two such studies (i.e., showing an identical point effect estimate) are in contrast. Yet, such errors are very common in the biomedical literature. Despite its seeming simplicity, the *p*-value is probably “*the most misunderstood, misinterpreted, and occasionally miscalculated index in all of biomedical research*” [15]. 

Driven by increasing concern, the American Statistical Association (ASA) has issued a statement [1] with six principles to guide the use and interpretation of *p*-values, and improve the conduct of quantitative science:

(1) “*p-values can indicate how incompatible the data are with a specified statistical model.*” Based on the observed data, the researcher either rejects or “fails to reject” the null hypothesis.
(2) “*p-values do not measure the probability that the studied hypothesis is true, or the probability that the data were produced by random chance alone.*” They can only tell us how consistent the data are with the null hypothesis.
(3) “*Scientific conclusions and business or policy decisions should not be based only on whether a p-value passes a specific threshold.*” Decision-making must not rest solely on a *p*-value. Researchers should also consider a broad range of other factors, e.g., study design, data quality, related prior evidence, plausibility of mechanism, real-world costs and benefits, etc.
(4) “*Proper inference requires full reporting and transparency.*” Researchers should not conduct multiple, unplanned analyses of the data and (selectively) report only those with certain *p*-values (i.e., those reaching statistical significance). This is usually called cherry-picking, data-dredging, significance-chasing, significance-questing, selective-inference, or *p*-hacking, and leads to a spurious excess of statistically significant results in the biomedical literature.
(5) “*A p-value, or statistical significance, does not measure the size of an effect or the importance of a result.*” For example, smaller *p*-values do not necessarily imply the presence of larger or more important effects, and larger *p*-values do not imply a lack of effect or lack of importance.
(6) “*By itself, a p-value does not provide a good measure of evidence regarding a model or hypothesis.*” Analysis of the data should not end with the calculation of a *p*-value. Depending on the context, *p*-values can be supplemented by other measures of evidence that can more directly address the effect size and its associated uncertainty (e.g., effect size estimates, confidence and prediction intervals, likelihood ratios, or graphical representations).

The ASA’s statement [1] outlined above discusses the main issues regarding the use and interpretation of *p*-values in applied research. Importantly, it also provides great references and supplementary material for anyone who would like to delve further into this topic. 

A comprehensive understanding of statistical concepts improves the quality of clinical research. We strongly encourage clinical researchers to involve biostatisticians and methodologists early on in their studies; to ensure a sound study design and a cohesive data collection strategy to minimize biases; to prespecify a data analysis plan that can address potential misuse and misinterpretation of *p*-values [16]; and to make use of appropriate guidelines for reporting biomedical research [17]. 

## Figures and Tables

**Table 1:** Results of the single-center, randomized, double-blind, placebo-controlled trial.

	Treatment Group		
Deaths	Active Drug	Placebo	*Total*
Yes	28	40	68
No	172	160	332
*Total*	200	200	400
Proportion of deaths	14%	20%	17%

Notes: *p*-value = 0.11, by a Chi-square test; Risk Ratio = 0.70, 95% CI: 0.45–1.09.

**Table 2:** Results of the multi-center, randomized, double-blind, placebo-controlled trial.

	Treatment Group		
Deaths	Active Drug	Placebo	*Total*
Yes	119	170	289
No	731	680	1411
*Total*	850	850	1700
Proportion of deaths	14%	20%	17%

Notes: *p*-value = 0.001, by a Chi-square test; Risk Ratio = 0.70, 95% CI: 0.57–0.87.
